# Supplementary material for: Are carbon emissions trading and green financial instruments synergistic? -Comprehensive quantitative research based on content analysis
Source: PLoS One. 2024 Mar 7;19(3):e0298601. doi: 10.1371/journal.pone.0298601 (PMC10919601; doi:10.1371/journal.pone.0298601)
Supplement: S1 Data — (ZIP) [file pone.0298601.s001.zip › Data/地级市Z_F工作报告.zip!/╔╜╬≈/╨├╓▌╩╨/└┤╫╘╣1⁄2╓┌║┼í╛┬φ┐╦╩2╛▌═°í┐_2018.docx]

各位代表：

　　现在，我代表市人民政府向大会报告工作，请予审议，并请市政协委员和其他列席会议人员提出意见。

　　一、2017年工作回顾

　　2017年，是忻州经济社会发展攻坚克难、奋力拼搏的一年。面对群众对美好生活需要的期盼，面对复杂多变的下行压力，我们坚持以习近平新时代中国特色社会主义思想为指导，在省委、省政府和市委的坚强领导下，认真贯彻落实习近平总书记视察山西重要讲话精神，牢牢把握稳中求进工作总基调，瞄准“三大目标”，坚守转型为纲，全面实施 “1661”发展战略，统筹推进稳增长、促改革、调结构、惠民生、防风险各项工作。

　　（一）经济增长回归合理区间。始终坚持“产业第一、项目至上、企业为重、服务为本”，把发展作为第一要务，着力推动经济平稳健康发展。

　　稳的基础得到巩固。完善固定资产投资分析调度会议、工业经济运行分析会议制度，建立县（市、区）长例会制度，集中研究分析经济运行情况，加强重点产业、企业、产品运行监测，及时解决具体问题。工业企业开工率提高到85%，规上工业企业实现利税比上年翻一番，28户企业 “小升规”，12户企业认定为省级“专精特新”企业。

　　进的态势逐渐强劲。牢固树立抓项目就是抓投资调结构、促转型惠民生、谋未来铸希望的理念，认真落实“三个主体”责任，96项省市重点工程全部开复工，继续坚持产业项目分类考核，新开工重大产业项目109个，总投资445.91亿元，完成投资占固定资产投资的30%以上。成立市、县招商服务中心，谋求融入环渤海经济圈，盯住长三角、珠三角，设立11个招商联络处，聘请19名招商大使；开展 “晋才晋商回乡创业创新”、“迎老乡、回故乡、建家乡”等招商活动。持续做好策划、储备、签约、落地、开工、建设、见效工作。储备项目1209个，总投资17664亿元，完成任务的133.8%；签约项目224个，总投资1427.68亿元，完成任务的189%；新签约项目开工103个，到位资金461.56亿元，完成任务的166%。

　　忧的瓶颈不断破解。持续在“减、免、缓、帮、扶、替”上下功夫，全力服务企业生产经营。实行重大项目领导包联责任制，推进入企服务常态化。及时帮助企业落实土地、电、水、气、热、网等要素。投运五寨500千伏超高压输电线路。认真解决环评、资金、环境、用工等问题，及时查处了保德中部引黄、五台山机场高速连接线阻工问题，争取中央、省级资金增长77%，17个PPP项目纳入全省推介目录。

　　全市地区生产总值增长6.7%，固定资产投资增长6.5%，社会消费品零售总额增长7%，一般公共预算收入增长5.8%，城镇居民人均可支配收入增长6.2%，农村居民人均可支配收入增长8.0%，居民消费价格涨幅0.7%，城镇新增就业3.81万人，城镇登记失业率3.74%，预期性指标全部实现预期目标。

　　（二）经济发展质量正在改善。坚持以转型综改为统领、以加快发展为目标、以开发区为平台、以招商引资为重点、以营造“六最”营商环境为切入点，着力主动转型、创新转型、深度转型、全面转型。全市战略性新兴产业工业增加值增长6.7%，高技术产业增加值增长20.4%。

　　大力推进供给侧结构性改革。化解煤炭过剩产能，完成3座矿井关闭任务，退出产能150万吨；3座矿井纳入“僵尸企业”；推动22座煤矿近2000万吨产能进行置换；地方煤矿销售产值增长44.54%。出台鼓励农民工和农民进城购买新建商品房奖励政策，打通商品房、保障房和安置房转换通道，房地产市场运行平稳；推进“忻材忻用、忻用忻材”。加强政银企对接，全年信贷投放24.32亿元；提升“助保贷”“过桥资金”水平，企业应急周转资金累计支持43.93亿元。取消、停征、降低45项涉企收费，全市共减免税费14亿元，33户直供电企业节省电费0.7亿元，城区非居民用管道天然气价格降低0.12元/立方米。

　　能源革命取得明显进展。新能源基地建设加快推进，装机300万千瓦风电项目列入晋北风电基地规划，完成22个项目立项，当年开工、投产光伏发电项目19.39万千瓦；新能源建成装机近500万千瓦，形成了以风电为龙头、以光伏为基础、以水电为骨干和生物质能、垃圾、燃气发电并存的新格局。

　　文旅产业发展扎实推进。市财政注资1.5亿元设立文旅产业投资基金；忻州古城同袁家村、雁门关景区同银泰、老牛湾景区同普惠完成合资，五台山景区与银泰进行合作。市和重点县组建旅发委，五台山景区成立旅游警察大队和旅游工商分局，34个景区体制机制改革任务完成，全域旅游发展规划加快编制。K601/K602号列车冠名为“五台山号”，五台山景区顺利通过中央文明委复查并继续保留“全国文明单位”称号，雁门关景区荣升国家5A级景区并荣获“2017中国十大最受欢迎旅游目的地”，芦芽山景区荣获 “国内十佳旅游目的地”称号，老牛湾村入选“2017中国最美村镇50强”。忻州古城保护改造进展顺利，云中河景区房车营地开工，“心灵之舟”旅游品牌更加响亮。全市旅游总收入同比增长24.65%，突破400亿元。

　　转型发展格局正在形成。贯彻落实国发42号文件，明确六大领域62条178项重点任务，积极争取试点示范政策支持，打出转型综改系列政策“组合拳”。原平市成为国家首批采煤沉陷区治理搬迁工作试点，五寨县创建国家首批农村产业融合发展示范园，定襄县成为国家级法兰锻造产品技术性贸易措施研究评议基地、国家级绿色锻造产业示范基地；56个传统产业项目列入全省工业转型升级重点项目库，完成投资31.76亿元；金宇科林高岭土产能扩张到20万吨，德奥公司由电梯延伸到立体车库，昌茂石油建成汽运服务产业链，忻州浪潮云计算大数据中心开工，蓝宝石、5G芯片等高技术产业项目落地。全市服务业增加值增长7.5%。

　　现代特色农业加快发展。以建设“中国杂粮之都”产业融合示范区为契机，强化特色产业基地、科技创新、品牌培育、人才建设，狠抓物流集散、市场开拓、电商营销等重点工作，现代农业产业体系、生产体系、经营体系加快构建。杂粮、中药材等特色产业加速发展，城郊农业、休闲农业、“互联网+”等新业态推进有力，有机旱作农业八大工程全面实施，全省有机旱作农业现场推进会在忻府区召开。全年新认证“三品”农产品217个，超任务近4倍；粮食总产36.6亿斤，再创历史新高。

　　创新驱动势头强劲。国家法兰锻件产品质量监督检验中心建成，山西省杂粮产品质量检验中心批准筹建，山西天宝、同德化工成为工信部企业科技创新品牌培育试点企业，山西省大型铸锻件工程技术研究中心、忻州市漫游世纪科技孵化器等4家创新主体成为我市首批省级科技创新平台；3户省首批创新板挂牌企业在山西股权交易中心挂牌，新认定高新技术企业5户、省级企业技术中心1户、省级中小企业技术中心5户、市级企业技术中心12户，全市众创空间增长1.5倍。

　　（三）脱贫攻坚着力再战。坚持“3659”策略，扭住“把握标准、精准识别、对症下药、统筹推进、群众主体、改进工作”六个环节，以习总书记视察我市为新起点，攻坚深度贫困。全市退出贫困村640个、减贫13.15万人。

　　完善责任体系。出台25个专项扶贫行动计划，细化7方面38项超常规举措，开展“对标回头看，大干一百天”专项行动和“冬季攻坚行动”，市县两级财政预算安排脱贫攻坚专项资金同比增长187.15%。

　　推进整村搬迁。坚持“六环联动”，认真解决旧村处置、新村配套、利益衔接等问题。累计搬迁434个村、9037户、21371人，入住率65.89%，搬迁村销号279个村。

　　主攻产业发展。以“一县一业一园区、一村一品一主体”为抓手，推进1619个贫困村脱贫产业实现全覆盖，引导838个村与龙头企业建立结对帮扶机制，1374个村成立合作经济组织。89个村级光伏电站全部并网发电。

　　实施技能培训。培训建档立卡贫困劳动力17200人，打造了“忻州月嫂”、“五台瓦工”、“繁峙绣娘”、 “静乐裁缝”、“保德好司机”、“代县古建匠人”等地域劳务品牌。

　　加快整村提升。加快补上水、电、路、网等基础设施短板，大力提升学校、卫生室、文化站等公共服务，努力改善村容村貌。769处农村饮水安全巩固提升工程完工，升级一批农村电网，农村公路建设投资10亿元以上，贫困村危房改造和危险土窑洞改造竣工。

　　深化专项行动。 “五位一体”金融扶贫小额信贷达到17亿元；82万亩造林任务均由扶贫造林合作社承担，吸纳贫困人口近万人；健康扶贫“双签约”实现全覆盖，农村建档立卡贫困人口全部纳入医疗救助范围，因病致贫、因病返贫问题得到破解；实现“两线合一”。

　　（四）重点改革大力推进。坚持求实求是，扭住关键环节，不断探索创新，努力靠改革破难题、转动能、增活力。

　　推进开发区（产业园区）改革创新。规划建设1个国家级开发区、7个省级经济技术开发区、2个生态文化旅游开发区、4个现代农业产业开发区，开发区规划体系得以建立，管理体制得以理顺，“三制”改革完成。全市入区企业数量、经营（销售）收入、投资强度、当年实际利用省外直接投资、研究与试验发展经费五项转型升级指标均完成省考核任务。

　　引深国企改革。组建市国有资产经营公司，云河集团转型脱困进展顺利，市押运护卫中心和保安公司改革实施重组，云母厂改制启动，市铁合金厂、锻压机床厂与田森集团混改加快推进，完成 “三供一业”分离移交年度任务，忻通公司、原平汽修厂厂办大集体改革有序推进，全市正常生产经营的50户国有企业全部完成公司制改制。

　　深化商事制度改革。工商登记实现“十七证合一”，前置审批事项除法律和另有规定外全部改为后置审批事项，企业登记全程电子化领先全省，“双随机、一公开”监管实现全覆盖，信用体系加快构建，市场主体增长17.60%。

　　农村产权制度改革、投融资体制改革、综合行政执法体制改革成效明显。

　　（五）民生事业持续改善。认真兑现各项惠民利民政策，民生投入占到公共预算支出的80%以上，同比增长17.4%。

　　继续改善基础设施。大西高铁原平西站与韩原线连接线铺轨，忻州至雄安高铁列入雄安新区高铁规划，神岢高速路基完工，五台山机场连接线有序推进，机场航站楼改造收尾。忻州城区44条新建改造道路通车， “创园”成果提升，成功创建国家卫生城市和省级文明城市。新增集中供热面积286万平方米。九龙岗森林公园、云中河景区二期加快推进，“一河一湖两山三组团”生态康养园区开展规划，体育公园开园。全市棚户区改造超额完成年度任务；静乐县创建国家园林县城，五寨县、繁峙县创建省园林县城。全市市政基础设施投资37.47亿元。特色建筑风貌整治100个村，农户庭院美化、农村街巷整治、村庄净化绿化“三大工程”顺利实施。“八道四治四建”专项行动推进有力。

　　不断改进公共服务。开展“教育振兴年”行动，启动文旅学院筹备、技工学院建设，城区新建十四中、雁门小学、和平小学、龙岗小学等进展顺利，忻府区等6个县（区）义务教育均衡发展通过国家认定。居民基本公共卫生服务人均财政补助标准提高到50元，公立医院药品实现零差率销售，14个县（市、区）医疗集团挂牌。原平市、忻府区甄家庄村及代县新高乡等10个乡村开展省级健康城市、健康村建设试点。文化部恭王府博物馆驻山西忻州（静乐）传统工艺工作站成立，忻州文化遗产精品展示月活动在京举办，代县雁绣等7个项目入选第五批省级非遗保护名录。全民健身活动广泛开展。忻州市国民体质监测中心启用。省政府确定的六件民生实事全面完成。安全生产稳定向好，社会大局和谐稳定。审计、统计、物价和公积金管理等工作得到加强，档案、地方志工作取得新进展，妇女、儿童、青少年、老龄、残疾人、红十字、慈善优抚、外事侨务等工作取得新进步。

　　认真搞好生态保护。中央、省环保督察交办群众反映问题全部办结，大气、水、土壤污染防治“三大战役”深入开展。实施河长制，执行“双主任”制度，无劣V类水体，水质优良断面比例92.86%；营造林82.24万亩；万元GDP能耗下降3.81%，全市环境空气质量综合指数向好。

　　突出做好就业增收。转移农村劳动力3.95万人；就业困难人员就业4545人，573名下岗失业退役士兵（士官）实现就业，7532名“4050”人员、零就业家庭、就业困难高校毕业生通过公益性岗位就业。企业退休人员、机关事业单位退休人员养老金标准提高；严格落实最低工资标准。公益性岗位人员工资、在职企业军转干部和退休企业军转干部生活困难补贴标准提高；农村居民经营性、工资性、转移性、财产性收入增速持续快于城镇居民。

　　着力提升社会保障能力。城镇职工、城乡居民养老保险继续扩面提标，机关事业单位养老保险纳入新制度运行；城乡居民基本医疗保险制度全面整合，财政补助提高30元，在北京、上海、海南等地实现跨省异地就医直接结算；农村低保标准年人均提高252元，完善医疗救助和临时救助帮扶制度，落实特困人员救助供养政策，高龄老人补贴、失能老人补贴、困难残疾人生活补贴、重度残疾人护理补贴按标准发放，60个农村老年人日间照料中心投入运营。创建14个全国减灾示范社区、75个省级综合减灾示范社区，防灾减灾能力全面提升。

　　（六）“六型”政府建设深入推进。始终坚守“执政为民、敢为人先、群策群力、滴水石穿”政府精神，认真落实“牢记宗旨、甘当公仆，勤奋学习、刻苦工作，恪尽职守、敢讲真话，依法办事、从严治政，严于律己、清正廉洁” “五章约法”，努力建设法治、为民、服务、效能、诚信、廉洁政府。

　　全面加强政治建设。坚持以习近平新时代中国特色社会主义思想为指导，深入学习贯彻党的十九大精神，切实增强“四个意识”、坚定“四个自信”，自觉维护以习近平同志为核心的党中央权威和集中统一领导，开展“维护核心见诸行动”主题教育，不断推进“两学一做”学习教育常态化制度化，认真贯彻落实省委、省政府和市委决策部署，坚持一切在大局下行动。

　　持续加强作风建设。认真落实政府党组全面从严治党主体责任和中央八项规定精神，严格执行国务院“约法三章”，持之以恒纠“四风”。注重政府干部的心理疏导和教育引导，自觉理政、谋政、勤政、善政、廉政，勇于担责、担诺、担急、担难、担险、担远，努力保持良好精神状态和工作状态。

　　全面推进依法行政。全面落实政府法律顾问制度，自觉运用法治思维和法治手段解决问题、推动工作。认真落实行政执法责任制。加强行政调解、行政复议工作。严格执行市人大及其常委会的决定，主动接受市人大法律监督、市政协民主监督和社会舆论监督。省人大、省政协建议提案9件、市人大代表建议210件、市政协委员提案326件全部依法及时办理。

　　加快转变政府职能。深入推进“放管服效”改革，把该放的真正放到位，把政府该管的坚决管好。取消、下放、调整市级行政审批等事项155项，清理规范8项行政审批中介服务事项。实施政府部门内设机构“大科室”改革，事业单位分类改革加快推进。在忻州经济开发区和原平市、宁武县、保德县试行企业投资项目无审批承诺制。

　　着力优化政务服务。推进“互联网+政务服务”，拓展“一网两平台”服务功能。建成运行覆盖市县乡三级互联互通的“13710”电子督办平台。深入开展减证便民专项行动，取消22%的申报材料。建立行政事业性收费目录清单制度，做到了目录之外无政府定价。

　　这些成绩的取得，是习近平新时代中国特色社会主义思想科学指导的结果，是省委、省政府和市委坚强领导的结果，是市人大、市政协有效监督、大力支持的结果，是全市干部群众同心同德、不懈努力的结果。在此，我代表市人民政府，向全市人民，向各民主党派、工商联和无党派人士，向各位人大代表、政协委员，向驻忻人民解放军、武警官兵、公安民警和驻忻单位，向所有关心、支持和参与忻州改革发展进步的同志们、朋友们，表示崇高的敬意和衷心的感谢！

　　各位代表，当前，忻州正处于转型升级的重要历史关头。必须清醒地认识到，我们的思想观念仍然不够解放，对转型升级的认识存在偏差；发展不平衡不充分问题严重，发展的质量和效益不高，长期积累的结构性矛盾远未根本解决；市场主体发育不充分，国有企业竞争力还不够强，民营企业发展缓慢，特别是大企业、好企业不多，转型发展的产业基础薄弱；传统产业转型升级尚处于初始阶段，新兴产业尚未成长壮大，新旧动能转换不快；城镇化水平不高，产城融合不够，中心城区带动能力不强；吸引新型产业落地的要素支撑乏力，尤其是高素质、专业型人才缺乏；创新创业动力不足，富有当地特色和市场意识的企业家队伍还不够壮大，远未形成共谋转型发展的浓厚氛围和竞进态势；农业现代化水平不高，脱贫攻坚任务艰巨，民生事业欠账较多，环境治理压力很大；政府职能转变还不够到位，不少干部的开拓意识不强、工作标准不高、担当精神不够。目前，忻州既需要转型升级，更需要实现跨越；既需要狠抓项目建设，更需要打造最优营商环境；既需要开启干部心智、激发创新活力，更需要强化法治观念，规范工作秩序；既需要促进发展，更需要安全稳定。

　　我们必须牢固树立机遇意识，把转型升级作为忻州发展的重大历史机遇，着力强化忻州正处于转型发展重大历史关头的认识，着力造就事在人为、奋发有为的导向，以对忻州长远发展和人民高度负责的态度，坚持问题起点、目标导向，靠苦功夫、笨办法，孜孜以求、久久为功，努力探索一条贫困落后地区在内乏活力、外有制约的情况下转型升级的新型道路，最终实现后来居上！

　　二、2018年工作安排

　　今年是贯彻落实党的十九大精神的开局之年，是改革开放40周年，是决胜全面建成小康社会、实施“十三五”规划承上启下的关键一年。做好今年的政府工作，要全面贯彻党的十九大精神，以习近平新时代中国特色社会主义思想为指导，进一步落实习近平总书记视察山西重要讲话精神，认真贯彻省委“一个指引、两手硬”工作总要求，认真落实省政府转型升级系列决策部署，自觉坚持市委对政府工作的统一领导，全面贯彻“1661”发展战略，大力推进改革开放，全面推动质量变革、效率变革、动力变革，统筹推进稳增长、促改革、调结构、惠民生、防风险各项工作，聚焦六大主战场主阵地，打好防范化解重大风险、精准脱贫、污染防治攻坚战，建设资源型经济转型发展“示范区”，打造能源革命“排头兵”，构建内陆地区对外开放“新高地”，提高保障和改善民生水平，促进经济社会持续健康发展，努力谱写新时代中国特色社会主义忻州篇章。

　　主要预期指标是：GDP增长6.5%左右，固定资产投资增长9%左右，社会消费品零售总额增长7%左右，一般公共预算收入增长2.38%，城乡居民人均可支配收入分别增长6.5%和7%以上，居民消费价格涨幅、城镇登记失业率、城镇调查失业率分别控制在3%左右、4.2%以内和6%以内。

　　约束性指标是：农村贫困人口脱贫12.1万人，城镇棚户区改造6022套，达到或好于Ⅲ类水体比例为78.57%、劣V类水体比例为0%，万元GDP能耗下降3.2%，完成省下达的其他环境质量改善指标和主要污染物总量减排指标任务。

　　完善转型升级考核评价指标体系，强化转型发展考核，更好发挥其“指挥棒”和“风向标”作用。

　　以上目标是按照高质量发展要求，立足我市阶段性特征，综合考虑需要和可能确定的，既把握发展第一要务要求，同时又为转型发展和高质量发展留足了空间。固定资产投资增长9%，主要考虑今年是全市“转型项目建设年”，要在确保投资增速高于经济增速的基础上，把更多注意力放在优化投资结构和提高投资有效性上。一般公共预算收入增长2.38%，低于全省4.12个百分点，主要是传统产业受价格影响增收乏力，新兴产业尚未形成骨干税源，减税降费后财政性增收放缓。农村常住居民人均可支配收入增长7%以上，高于省0.5个百分点，是要在保持城乡居民人均可支配收入与GDP同步增长的同时，实现农村居民人均可支配收入增长快于城镇居民人均可支配收入增长的目标。今年重点做好以下10个方面工作：

　　（一）坚持以习近平新时代中国特色社会主义思想为指导，着力建设引领转型升级的先进思想观念

　　深入学习习近平新时代中国特色社会主义思想。把之作为推动忻州转型升级和高质量发展的强大思想武器，转化成为全市干部群众的自觉行动和根本追求。

　　发挥思想文化引领作用。坚定落后地区改革完全可以一步到位的自信，在全市厚植忻州转型升级一定能够发挥后发优势、实现奋勇争先的思想观念，大力弘扬忻州的传统文化，特别是睁眼看世界、敢为天下先的徐继畲文化，艰苦创业、以弱胜强的根据地文化，雷厉风行、开拓创新的走西口文化，不辱使命、群策群力的杨家将文化，毫不利己、专门利人的白求恩文化，坚守事在人为、主动作为、奋发有为的坚定信念和不懈追求。

　　推动思想观念与时俱进。围绕纪念改革开放四十周年，大力推进思想解放，争当自觉践行转型升级思想观念的先进典型，带动全市干部群众不断打开心门，树立开放、市场、竞争、法治、创新、诚信的观念，用地球村眼光看问题，用市场手段调结构，用法治思维、法治手段破难题，在不断超越自己中实现人无我有、人有我优、后来居上。

　　打造转型升级的新的语言环境。充分运用主流媒体、新媒体、大讲堂、培训班等有效形式，切实让“开发区建设”、“招商引资”、“‘六最’营商环境”、“岸港网”、“大数据”、“信息化”、“改革创新”、“技术变革”、“文旅产业”等成为干部群众日常生活最集中的话题，营造浓厚的语言环境，并内化成干部群众的核心理念、自觉意识、行为习惯，推动忻州各项事业的全面发展。

　　（二）着力引深脱贫攻坚战，认真实施乡村振兴战略，大力提升城镇化水平，主攻区域经济发展

　　在习总书记视察忻州一周年之际，进一步兴起学习总书记视察山西重要讲话精神和深度贫困地区脱贫攻坚座谈会讲话精神新高潮，对贯彻落实总书记指示进行再动员，不断引深脱贫攻坚战，为乡村振兴奠定坚实基础，着力加快区域经济社会事业的发展。

　　提高脱贫质量。在抓基本、夯基础、见成效上下功夫，切实弄通基本政策、吃透基本情况、掌握基本力量、打好工作底色、培育工作骨干、做实基本工作，最终把工作落到“易地搬迁任务完成、产业发展明显进步、内生动力大幅增强、村庄设施根本改变、群众满意度跃升、长远发展体制机制建立健全”六个标准上，坚持效果为王，以实绩论英雄，坚决打好精准脱贫攻坚战。

　　攻克易地搬迁。整村搬迁203个村、5747户、13790人，做到人搬出、房拆除、地复垦，新村设施建设配套、小区管理完善、后续产业发展，“人走在哪、利益调整在哪”，保障好搬迁群众的基本利益，扩大在新村的利益。加快整村提升，与乡村振兴、人居环境改善、特色风貌整治有机结合，优先安排群众最期盼的项目、优先解决群众反映最强烈的问题。

　　扭住产业发展。坚持以户为核心、以合作社为基础、以乡村为依托、以产业为抓手、以政策为杠杆、以发展为根本，抓好家长、社长、乡村长“三长”和产业体系、政策体系、品牌体系“三个体系”，为群众稳定脱贫提供坚实支撑。全力推进光伏扶贫项目，为每个贫困户稳定增收3000元创造机会；实施退耕还林、生态治理、生态保护、经济林提质增效、特色林产业“五个一批”林业生态扶贫项目互联互动，让更多贫困人口获得更多劳务收入；加大电商扶贫力度，巩固完善4个全国电子商务进农村综合示范县建设成果，争取再有2个国家级贫困县列入示范县。实施特色产业扶贫工程，努力使60%左右的贫困人口通过产业扶贫实现脱贫。

　　攻坚深度贫困。认真落实教育扶贫、健康扶贫、金融扶贫等各项政策，发挥好社会保障政策兜底作用。继续搞好农村贫困劳动力免费职业技能培训，提升区域特色劳务品牌，提高贫困群众就业能力。主攻繁峙、神池、五寨、岢岚、河曲、保德6个县摘帽，为其他县2019年脱贫奠定基础。

　　提升治理能力。深化乡村治理，加快构建以党支部为核心、以村委会为依托、以经济合作组织为基础，各专业协会、社团组织积极配合的乡村治理新格局。加强对群众的教育引导，通过制定村规民约、提升村民素质、改善环境卫生、推动邻里互助，为实现产业兴旺、生态宜居、乡风文明、治理有效、生活富裕而不懈努力。

　　不断优化布局。以易地扶贫搬迁为契机，切实搞好乡村撤并、整合，推动形成以忻定原城市群为核心、以各县城为区域中心、以重点乡村和生产经营活动点为基础的新型城乡格局。今年城镇化率再提高1.5个百分点以上。启动定襄县撤县设区、忻府区3个乡镇改街道办事处。

　　培育特色农业。加快以杂粮为重点的特色农业产业发展，支持神池县建设全省有机旱作农业示范县，实施忻府区辣椒等5个全省有机旱作农业封闭示范片建设。以工业化理念、产业化路子推动农业内部三次产业分工，扶持原平石鼓等杂粮加工企业发展，提升农产品加工业水平。加大“三品一标”认证力度，叫响“粮忻”产品品牌，擦亮“中国杂粮之都”名片。推动农业和加工、服务、旅游、文化、健康、养生产业结合，加快一二三产融合发展。大力推进忻定原城郊农业发展。加强耕地综合生产能力建设，推进水利重点工程建设，提升农业机械化水平。认真落实粮食安全责任，做到“藏粮于地、藏粮于技”。

　　大力改进作风。扎实开展“脱贫攻坚作风建设年”，继续引深“学习刘桂珍先进事迹”活动，持续开展扶贫领域腐败和作风问题专项治理，切实压实各级干部的主体责任，坚持奖罚分明，搞好政策转化，提振干部精神状态，不断激发群众内生动力，使 “我要脱贫”成为广大群众的共同追求。

　　（三）深化对忻州转型升级的规律性认识，着力做好高质量增长的基层基础工作，争当能源革命“排头兵”

　　确立“三步走”转型目标。不断完善转型升级的总体规划，切实形成完整的规划体系。到2020年，激活传统产业存量，培育新兴产业增量；到2025年，实现二者并驾齐驱，新兴产业比重占到50%以上；到2030年，战略性新兴产业成为全市增长的主力，完成转型升级的目标。在实现时间上与全省保持同步，在具体步骤上体现忻州实际。

　　构建现代产业体系。推动现有产业转型升级，靠扩张、延伸、提升、衍生促转化。推动市场需求转化成新兴产业，围绕旅游、康养、绿色等消费升级需求，通过挖掘、捕捉、激发积极要素，不断把潜在需求转化成现实产业。推动区位优势转化成优势产业，不断壮大文化旅游、新能源、新材料、现代煤化工、现代装备制造、节能环保、现代服务业、现代特色农业、新一代信息技术产业九大战略性新兴产业。设立6000万元市级技改资金支持传统产业改造提升；围绕每个产业，认真布局产业链、构建利益链、培育要素链、组织供应链、配备销售链、优化创新链、制定政策链、建设人才链。大力发展民营经济，聚焦产业、企业、企业家，大力弘扬和保护企业家精神，推动 “个转企、小升规、规改股、股上市”，新注册企业1000户以上，新培育“小升规”民营企业15个、省级“专精特新”企业10户以上，推动规范化股份制改造民营企业10户以上。

　　争当能源革命排头兵。推动煤炭减量置换和减量重组，确保晋能保德低热值煤电厂投产，推进河曲桃园低热值煤发电项目开工，促进煤炭绿色低碳高效开发利用，提升煤炭先进生产力占比，延伸煤炭产业链条。加快新型能源基地建设，新增风电装机100万千瓦，建成投运8个集中式光伏扶贫电站、1350个村级电站。支持繁峙奥博、河曲燠晶硅等光伏组件企业发展壮大，推动光伏产业全产业链发展。积极发展绿色载能产业，推动国电投山西铝业铝镁合金一体化改革。深化煤层气开发利用，加快重点用户、重点旅游区和重点城镇的管线建设，形成覆盖全市的大燃气网。

　　强化质量标准改革。设立市长质量奖，开展质量专项行动，提升国家级法兰锻件产品质量监督检验中心水平，加快省级杂粮产品质量检验中心建设，充分发挥标准提升对质量变革的引领和倒逼作用，助推产业提质升级。

　　（四）引深开发区改革创新发展，着力造就集聚发展、转型发展、创新发展的竞进态势，创建综改“示范区”

　　加快分类推进，形成竞进格局。积极支持忻州经济开发区建成国家级开发区，繁峙、静乐、定襄、河曲工业园区获批省级开发区，推动其余县工业园区申报省级开发区，建设代县、偏关2个文化生态旅游开发区和五台、静乐、五寨、神池4个现代农业产业开发区，使忻州经济开发区、五台山景区、忻府区代表忻州发展高度，原平等6个县（市）支撑起全市发展基本面，静乐等7个县充分彰显忻州特色，着力在全市形成依托开发区转型升级的竞进态势和争相发展的良好局面。

　　坚持区别对待，强化分类考核。把项目签约、落地、开工、投产、运行作为开发区工作的考核标准，强化投入产出效益考核，切实提升园区承载力，大力提高工业项目投资强度、单位面积产出率和税收贡献率。忻州经济开发区至少新建5个投资超10亿元的转型项目，原平经济技术开发区至少新建5个投资超5亿元的转型项目，其他开发区（园区）至少新建3个投资超2亿元的转型项目。

　　搞好起步区建设，狠抓首位产业发展。认真实施开发区改革创新发展体系规划，着力推动新兴产业向开发区集聚、新技术项目向开发区集中。优先在开发区布局转型项目，工业项目原则上全部入园进区。创新开发区基础设施投融资模式，搞好公共服务平台建设。加快“五规合一”，搞好“九通一平”，变“项目等地”为“地等项目”。忻州经济开发区力争在都航小镇开工、蓝宝石项目投产、标准化厂房和人才公寓建设等重点工作上取得突破，彰显转型升级的示范效应。要充分发挥忻州大学生双创基地作用，深入开展“五小”活动，激发更多创客投身创业创新。

　　落实“三化三制”，深化体制机制创新。巩固忻州经济开发区、原平经济技术开发区 “三化三制”改革成果，总结经验、汲取教训，不断深化各项改革措施在各类园区的落实。理顺开发区（产业园区）与属地的关系，切实向开发区（产业园区）放权。落实好支持开发区的各项政策，充分发挥开发区建设引导资金的作用，切实撬动社会资本，真正把开发区（产业园区）打造成忻州转型升级的主引擎。

　　（五）以“铁公机”“岸港网”为先导，以文旅产业为平台，打造内陆地区对外开放的“新高地”

　　持续改善基础设施，大力发展“铁公机”“岸港网”。加快推动忻州至雄安高铁前期，确保原平至太原动车组8月1日通车。推动繁五高速开工，完工五台山机场连接线、神岢高速、晋蒙黄河大桥，加快“三大板块”旅游公路和“四好农村路”建设，做好108线、209线、338线等国省道的改造提升工作。完成五台山机场航站楼国际区改造，推动繁峙通用机场启动建设，争取原平通用机场项目获批，加快代县通用机场前期工作。发挥定襄永旺物流园区出口监管仓库和保税仓库作用。推进政务云、工业云大数据产业，完成浪潮忻州云计算中心建设，努力提高产业转型升级的信息化水平。完成1475个行政村光纤宽带网络建设和65个行政村光纤宽带网络改造。

　　充分发挥文化旅游战略支柱产业和对外开放大平台的巨大作用。全力实施黄河、长城、太行三大旅游板块突破性开局行动，扎实推进旅游“重点项目开工、重大战投引入、重点景区提升”工作。不断深化景区体制机制改革，加快旅游产业发展、产品开发。深化五台山景区的开放合作，加快芦芽山景区的改革，拓展雁门关、老牛湾等景区的改革成果，基本完成秀容古城保护性开发，加大对奇村、顿村、合索温泉的开发力度。推动芦芽山景区冲刺国家5A级旅游景区，偏关老牛湾、静乐天柱山、原平天涯山、繁峙滹源景区、河曲临隩公园、代县文庙创建国家4A级旅游景区；办好 “最美冰洞、世界奇观”系列推介和“看黄河、走长城、穿越太行”越野自驾游等活动；推进景区“厕所革命”；投运云中河景区房车营地和婚礼广场；推进国家全域旅游示范区创建工作和 “一河一湖两山三组团”生态康养景区、旅游集散中心等旅游重点项目，加快忻州旅游产业化、特色化、品牌化步伐。

　　继续打造“三个门户、三个集散地、六大经济板块”，切实构建内陆地区对外开放 “新高地”。抓住改善优化软硬条件、项目引进、招商引资等关键环节，加快提升三个对外开放门户、三个集散地的功能，真正使忻州成为西部进入京津冀的桥头堡、京津冀休闲康养的后花园。以产业园区为载体，健全产业链为关键，提高项目集聚集约程度为重点，进一步加快 “六大经济板块”建设，真正使忻州成为人流、物流、资金流、信息流、人才流的新凹地，对外开放的“新高地”。

　　（六）强力推进重点领域改革，努力打造“六最”营商环境，不断培育发展新动能

　　深化供给侧结构性改革。继续在“减、免、缓、帮、扶、替”上下功夫，进一步降低企业制度性交易成本和要素成本，力争全年新增电力直接交易企业12户以上。关闭恒腾煤业等3座矿井。加大“忻材忻用”力度。做好房地产去库存工作。

　　深化企业改革。坚持“一企一策”，通过做强一批、整合一批、重组一批、转型一批、股改一批、注销一批、破产一批、划转一批、探索一批推进国企改革。深化市国有资产经营有限公司改革，完善市保安押运护卫中心法人治理结构。市锻压机床厂、铁合金厂与田森集团战略合作取得实质性进展。加快市云母材料厂、酒厂改革。全面完成市县两级国有企业 “三供一业” 移交、企业办社会职能分离、厂办大集体改革任务。推动民营企业加快建立现代企业制度，实现由家族式、小作坊向现代企业的跨越。

　　深化金融改革。完善政银企对接机制，加快推进金融产品创新，力争银行信贷全年新增100亿元以上。引导险资入忻，推广人保财险“政融保”融资项目，争取年内实现保险资金支农支小3亿元。做大做强市县两级融资平台。加大企业上市帮扶力度。推动德奥产业基金落地运营。做大融资担保。启动宁武等5个县农信社改制农商行。

　　深化农村改革。完成承包地确权登记颁证工作，完善承包地、宅基地 “三权分置”制度。培育壮大各类新型经营主体。深化农村集体产权制度改革，探索“资源变资产、资金变股金、农民变股东、收益有分红”的有效途径。培育打造30个以上农业产业化联合体。完善国有林场改革，深化集体林权制度改革，搞好集体公益林委托国有林场管理试点。加快推进供销合作社综合改革。

　　深化“双创”战略。认真实施人才体制机制改革，大力倡导“人人都是人才、人人可以成才”的理念，不断激发转型升级的内生动力和强大活力。积极培育一批低成本、便利化、全要素、开放式、专业化众创空间，力争年底省级以上众创空间达到10家以上。支持永旺创业园创建省级科技企业孵化器，新建2家以上省级以上农村“星创天地”。引进高端人才，培育乡土人才，用好专业技术人才，有力促进各项事业发展。

　　深化“1331”战略。支持忻州师范学院创建全省一流本科院校，启动忻州师范学院新校区建设。鼓励企业建设技术中心。新建1家省级重点实验室，新增2家高新技术企业。通过学前教育抓覆盖、义务教育抓均衡、高中教育抓提升、技工教育抓发展、职业教育抓特色、大学教育抓转型，不断优化素质教育体系和职业教育体系。

　　优化营商环境。引深“1+10”专项行动，全市域推行企业投资项目承诺制。对政府部门既及时部署、又带头示范，认真为企业搞好服务；对垄断企业既大力支持、又严格监管，在改进服务中拓市场、增收入；对中介组织既积极培育、又有效规范，充分发挥法律服务、会计审计、环境评估等社会中介机构作用；对企业家既依法严管、又尊重关心，真正使企业家成为忻州转型发展的发动机和主引擎；对员工既要激发自身活力、又要加强教育培训，让“工匠精神”内化于心、外化于行；对群众既高度关心、又积极引导，在全市造就为自己生活的地方富起来、强起来而共同努力的良好导向和浓厚氛围。

　　（七）紧紧扭住项目建设不放松，深入开展“转型项目建设年”活动

　　树立鲜明导向。以项目建设为主题，以转型项目为标志，以园区发展、集聚发展为主攻方向，坚持把抓项目、促转型作为工作常态，真正把全市干部群众的注意力和兴奋点凝聚到抓好项目建设、促进转型升级上来，力争使我市“转型项目建设年”工作走在全省前列，为忻州的转型发展、后来居上奠定基础。

　　聚焦“四个一批”。集中策划、包装、储备一批。在3月底前市、县建成项目库；全年储备项目计划总投资超过2017年全市固定资产投资实际完成额的2.5倍以上；转型项目（主要包括新兴产业、传统产业改造升级、科技创新项目）投资完成额占全年固定资产投资的60%左右；基础设施、生态保护与修复、脱贫攻坚等民生项目投资完成额占全年固定资产投资的40%左右。

　　集中招商、签约、入库一批。充分发挥市、县招商服务中心的作用，深入开展“晋才晋商回乡创业创新”主题招商活动，继续实施好 “迎老乡、回故乡、建家乡”签约项目，不断创新招商方式，积极开展产业模块化招商、产业链招商，力争在引进世界500强和国内500强上取得新突破。

　　集中落地、开工一批。开展好“前期手续集中办理月”和“项目集中开工月”活动，进工地、到一线、解难题，4月底前续建项目全部复工，新建项目30%以上开工。按照 “有意向的项目不签约不放松、签约的项目不注资不放松、注资的项目不开工不放松、开工的项目不投产不放松” “四个不放松”的要求，A类县分别落地、建设3个以上重大转型项目，B类县分别落地、建设2个以上重大转型项目，C类县分别落地、建设1个以上重大转型项目。

　　集中建成、投产一批。在确保工程质量和安全的前提下，抢工期、抓进度，形成尽可能多的实物工程量；对竣工验收事项并联办理、平行作业、一站式办理，打通投产前“最后一公里”，同步完成配套附属设施建设，推动项目早日达产达效。批复的光伏扶贫项目全部在上半年建成。

　　狠抓重点示范。实行县（市、区）长 “一园六企”制度，每位县（市、区）长联系1个开发区（产业园区）和一产、二产、三产各2个转型项目。市本级重点抓好“中国杂粮之都”产业融合核心园区建设、国家级杂粮产地交易市场建设、中国杂粮产业优势区创建、优质杂粮工程示范市创建、“好粮油”示范市创建，忻州古城开发保护改造、康养产业集群发展、云中河两岸开发、大西高铁站前广场活化等项目。实施好26项省级重点工程、80项市级重点工程。

　　优化工作机制。建立项目建设常态化推进六项工作机制，认真落实各县（市、区）政府、项目建设单位、各职能部门的“三个主体”责任。进一步搞好策划、做实储备、推动签约、狠抓落地、督促开工、加快建设、主攻见效，严格考核，强化督查，改进服务，切实破解土地、环评、资金、人才等制约，不断优化转型项目建设投运环境。

　　（八）大力加强生态文明建设，以环保倒逼转型升级和高质量增长

　　加快推进生态修复治理。实施林业十大生态修复工程，全面开展国土绿化，营造林36.5万亩以上。完成灵河高速通道绿化，提升五保高速、太佳高速、大运高速通道绿化成果。启动筹建忻州城区金银山城郊森林公园。新一轮退耕还林4.61万亩，发展特色经济林30万亩。细化河长制工作方案，压实各级河长责任，规范河流管理，实现河畅水清。推进汾河、滹沱河、桑干河、大青河等重点流域生态综合治理。开工建设汾河堤外湿地。扎实推进绿色矿山创建工作。

　　打赢污染防治攻坚战。深入开展大气污染防治行动，打赢蓝天保卫战。将禁煤区、禁燃区划定逐步扩大到忻府区、原平市、定襄县，大力推进集中供热，积极支持煤改气、煤改电等清洁能源替代工程；其他县全部划定禁煤区，并逐步扩大禁燃区范围。依法做好禁止燃放烟花爆竹、燃烧旺火工作。加强施工扬尘、汽车尾气、车辆运输等污染控制。强化工业企业深度治理，完善推进工业企业错峰生产。着力开展清水行动，打赢黑臭水体歼灭战。全面提升水环境质量；推进乡镇污水处理设施建设；实行地表水国考断面采测分离，加快建设5个地表水水质自动监测站。扎实推进净土行动，打赢土壤防治持久战。深入实施化肥农药使用零增长行动，大力推进畜禽养殖污染防治和秸秆综合利用，有效减少农业面源污染。着力巩固扩大中央、省环保督察工作成效，确保整改问题不再反弹。

　　推进资源能源集约节约利用。落实最严格水资源管理制度。优化水资源全域配置，严格控制“三条红线”，积极推进地下水水位和水量“双控体系”建设，强化工农业和城市生活节水，提高水资源利用效率和效益。高质量开展第三次土地调查，高标准编制重点规划，加强土地规划执行管理，强化节约集约用地考核；持续深入开展批而未用土地清理行动，加大闲置土地处置力度，不断提高供地率。实施能源总量和强度双控行动，强化重点领域节能管理，深入推进能效对标活动，大宗工业固废综合利用率达到67.5%。

　　压实生态环境监管责任。严格落实环保部门的综合监管责任、各职能部门的部门监管责任、各行业的行业监管责任、各类监测单位的专门监管责任、各级政府的属地监管责任，实现环境监管责任全覆盖。完善环境监管网格化管理，推动环境监管关口前移、触角下延。深化生态文明体制改革，积极争取在汾河、滹沱河、桑干河等跨市流域开展横向生态保护补偿试点工作。

　　（九）切实防范化解重大风险，认真办好社会安全稳定的每一件事

　　巩固安全生产局面。完善“4438”工作机制，落实“四铁”要求，加强隐患排查整治和风险管控，把安全生产隐患解决在未萌、未发状态。健全完善各项安全生产制度，着力推进安全生产标准化达标创建，严格按安全生产规程办事。深入开展安全生产专项整治行动，严厉查处违法违规非法生产。全面推进全国安全生产宣传教育“七进”活动试点市建设，切实打造安全生产的社会环境。实行重奖重罚，建立良好导向，坚决杜绝重特大事故，有效遏制较大事故，努力减少一般事故，着力提升本质安全水平。

　　防范化解重大风险。打好防范化解金融风险攻坚战，加强对网络信贷、投资公司等监管，规范类金融机构发展。严防严打非法集资、金融诈骗等违法活动，坚决守住不发生区域性金融风险底线。防范化解地方政府债务风险，严格政府债务管理，严禁各类违法违规举债、担保等行为。积极稳妥处置存量债务，健全规范的地方政府举债融资机制。

　　提升社会治理水平。深化平安忻州建设，完善立体化、信息化治安防控体系，依法开展扫黑除恶专项斗争，推动社会治理重心向基层下移。完善重大决策社会稳定风险评估机制，加强对社会热点难点问题的引导，依法解决群众合理诉求。加强食品药品安全监管。做好防灾减灾救灾工作。加强国防动员、人民防空、双拥共建和民兵预备役工作，推动妇女儿童、老龄、关心下一代、红十字会、慈善、残疾人、科普、气象、防震减灾、档案、外事、侨务、民族宗教等事业再上新台阶。

　　（十）扎实办好民生实事，让人民群众在共建共享中拥有更多的获得感和幸福感

　　推进创业就业。深化创业型城市创建活动，加大对农民工、高校毕业生和退役士兵等返乡下乡创业支持，加强创业孵化基地、创业园区建设。做好公益性岗位就业人员管理与开发工作，对就业困难高校毕业生提供重点就业援助，确保零就业家庭即时动态消零。扎实做好退役军人安置。深入推进“无欠薪市县”创建活动，构建和谐稳定劳动关系。

　　深化教育振兴。发展普惠性学前教育，扩大公办幼儿园和普惠性民办幼儿园覆盖率。推进义务教育均衡发展，静乐、偏关两县通过义务教育均衡发展省级验收和国家督导评估认定，定襄等7个县完成“全面改薄”项目年度任务。扎实做好消除大班额工作。改善高中办学条件，做好新高考三项试点。大力发展现代化职业教育，全面完成县级职教中心建设任务。实施“县管校聘”，加强教师队伍建设。推进基础教育教学创新，缩小城乡、校际差距，有效解决中小学课外负担重、“择校热”等突出问题。继续改进中小学课后服务工作，减轻学生过重课业负担。

　　加强全民健康。持续深化医疗卫生机构一体化改革，实施“136”兴医工程。健全科学合理的分级诊疗制度，做好家庭医生签约服务。巩固取消药品加成和医疗服务价格改革成果，健全完善公立医院现代管理运行新机制。实施国家基本和重大公共卫生服务项目，深入开展爱国卫生运动，推动健康城镇创建。改进医疗服务，提升医疗水平。切实保护医护人员的合法权益，依法严打“医闹”、伤医事件。改善妇幼保健服务。加强全科医生队伍建设。加快发展中医药事业。鼓励规范社会办医。加快推进医养结合。推动全民健身和全民健康深度融合。投运市儿童医院和市残疾人康复托养中心。

　　强化文化服务。弘扬中华民族优秀传统文化，继承革命文化，发展先进文化，培育和践行社会主义核心价值观。深入实施文化惠民、文化传承工程。大力发展文化体育产业，深入开展全民健身活动，不断满足人民群众的精神文化需求。加强文化遗产和文物保护利用。强化文化市场监管，净化网络环境。办好非物质文化遗产博览“长城系列”活动，做好全国第二届青年运动会拳击比赛筹办工作。全面完成市县方志年鉴编纂任务。

　　完善社会保障。实施全民参保计划。推进机关事业单位编制内人员参加养老保险。健全被征地农民基本养老保险制度。落实建档立卡贫困人口、低保对象、特困人员等困难群体养老保险代缴政策。加快省属国有煤炭企业医疗、生育保险纳入属地社会统筹步伐，提升失业、工伤、生育保险规定职业人群覆盖率，实现社会保障由制度全覆盖到法定人群全覆盖。扩大跨省异地就医直接结算范围，全面落实农村建档立卡贫困人口“三保险、三救助”医疗保障帮扶政策。提高失业保险金标准和城乡低保标准。健全农村留守儿童和妇女、老年人关爱服务体系。

　　建设宜居环境。不断深化“五城联创”。巩固“创卫”、“创文”成果，创建国家文明城市、园林城市，推进智慧城市、环保城市创建。忻州城区以打通干线节点、方便居民出行，提升园区路网、促进产业振兴，大力补绿增绿、美化城市生态，完善基础设施、优化城市功能为重点，着力改善城市人居环境。继续改造和新修市政道路30条，以公园绿地服务半径500米规划街头小型游园、广场，合理规划社区公园、游园，满足市民生活、休闲需求；城市建成区12%以上的面积达到海绵城市目标要求；完工九龙岗森林公园和云中河景区二期工程。启动河曲、五台两县申报“国家园林县城”工作，全市新增园林绿化面积200万平方米。大力推进棚户区改造。加快城区市容环境卫生和便民市场等便民服务设施建设，推进生活垃圾焚烧处理设施改造，促进城市管理精细化、规范化、高效化。认真办好省政府确定的六件民生实事。

　　提高居民收入。加强企业工资分配宏观指导，实行以增加知识价值为导向的分配政策。引导土地承包经营权、林权有序流转，增加农民经营性和财产性收入。继续提高退休人员基本养老金和城乡居民基础养老金发放标准。规范机关事业单位工资和提高津补贴标准。

　　三、大力改进政府自身建设

　　在转型升级的重要历史关头，必须牢固树立“功成不必在我、但求无愧后人”的政绩观，有担当、敢作为、善成事，一心一意干事业，全心全意谋发展。

　　全面加强政治建设，坚持正确政治方向。牢固树立“四个意识”，始终坚定“四个自信”，自觉接受市委领导，自觉接受市人大、市政协以及社会各界的监督。切实引导好、管理好政府系统干部队伍的状态，坚持一切在大局下行动。着力打造引领转型升级、脱贫攻坚的思想观念，努力造就各部门、各县（市、区）争先发展的竞进态势和放心放手工作的良好氛围。

　　努力建设“六型政府”，始终坚守政府精神。持续建设法治、为民、服务、效能、诚信、廉洁 “六型”政府，不断弘扬“执政为民、敢为人先、群策群力、滴水石穿”的政府精神，把政府自身建设引向深入，更好服务、促进、保障忻州转型升级和高质量发展。

　　优化政府定向引导，切实用足用好政策。综合运用政策激励、考核引导等多种手段，发挥好财政奖补资金“四两拨千斤”的作用，为转型发展提供更精准的引导、更周到的服务。认真研究、运用各种文件、政策，找出同忻州的结合点，做到融会贯通、灵活运用，真正把文件精神转化成落实措施，把上级语言转化成地方语言，始终把握工作的主动权。

　　提升政务服务水平，全面提高政府效能。坚持“产业第一、项目至上、企业为重、服务为本”，着力为企业、群众和一切市场主体提供良好的政务服务。持续推进“放管服效”改革，完成全市电子政务“一网两平台”改造升级。全面实施市场准入负面清单制度，推进“证照分离”改革。全面实施“双随机、一公开”监管，深化、拓展、延伸“13710”工作制度，办好忻州“随手拍”问政平台，推进政府效能革命，更加自觉地理政、谋政、勤政、善政、廉政，担责、担诺、担远、担急、担难、担险。

　　全面推进依法行政，打造忻州诚信品牌。把严格依法办事作为政府最大的诚信品牌，做到一切行为在法律和政策范围内活动；高举严格依法办事和维护群众合法利益两面旗帜，大力倡导契约精神，在全市上下形成人人讲诚信、个个守信用的浓厚氛围。大力改进和加强政府监管，切实把政府该放的权放到位、该管的事管到位；严厉打击各类破坏市场秩序的行为，营造公平、公开、公正的良好竞争环境。自觉接受媒体监督，依法查处冒用、盗用记者身份进行敲诈的行为，切实维护各类媒体在忻州的良好形象。

　　全面加强政风建设，不断引深从严治政。认真落实全面从严治党要求，始终把纪律和规矩挺在前面，深入推进政府系统党风廉政建设和反腐败斗争，认真落实中央八项规定精神和市委实施意见，持续狠刹“四风”。坚持“紧日子、保基本、调结构、保战略”要求，厉行勤俭节约，切实把有限的资金用在最需要的地方。突出整治形式主义、官僚主义，严肃查处懒政怠政、推诿扯皮、弄虚作假等行为。旗帜鲜明地为勇于担负、敢于负责的干部撑腰做主，最大程度地激发全市上下投身转型发展的强大动力。

　　各位代表，新的征程已经启航。让我们更加紧密地团结在以习近平同志为核心的党中央周围，在省委、省政府和市委的坚强领导下，凝心聚力、真抓实干、攻坚克难，全面完成今年各项目标任务，为我们共同生活的地方发展态势越来越好、创业环境越来越优、人居环境越来越佳而努力工作！
